# Supplementary material for: Evolved histone tail regulates 53BP1 recruitment at damaged chromatin
Source: Nat Commun. 2024 May 31;15:4634. doi: 10.1038/s41467-024-49071-w (PMC11143218; doi:10.1038/s41467-024-49071-w)
Supplement: Supplementary file 1 — Supplementary Information [file 41467_2024_49071_MOESM1_ESM.pdf]

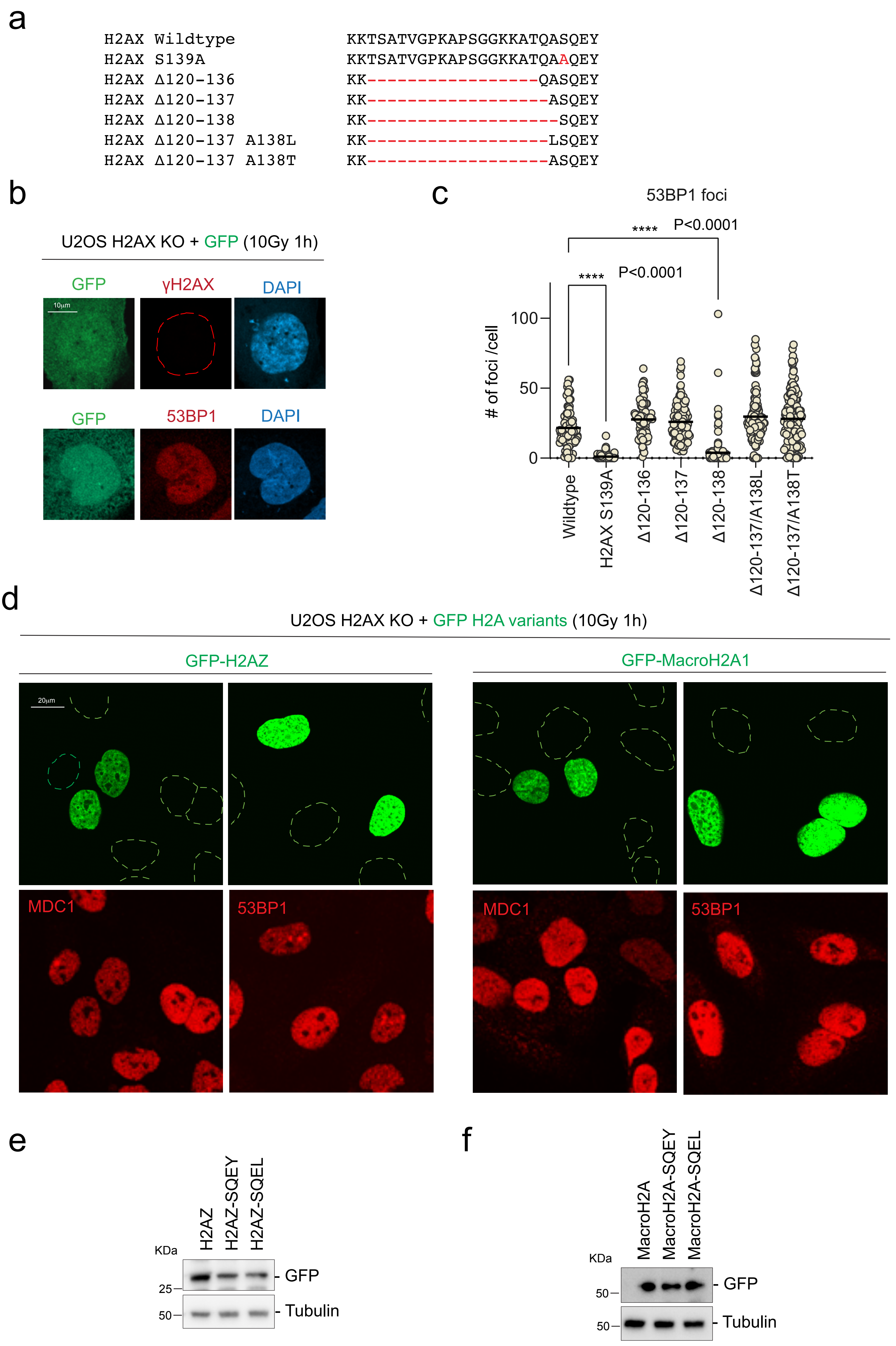
a
H2AX Wildtype KKTSATVGPKAPSGGKKATQASQEY
H2AX S139A KKTSATVGPKAPSGGKKATQAAQEY
H2AX Δ120-136 KK------------------QASQEY
H2AX Δ120-137 KK-------------------ASQEY
H2AX Δ120-138 KK--------------------SQEY
H2AX Δ120-137 A138L KK-------------------LSQEY
H2AX Δ120-137 A138T KK-------------------ASQEY
b
U2OS H2AX KO + GFP (10Gy 1h)
GFP
γH2AX
DAPI
10µm
GFP
53BP1
DAPI
c
53BP1 foci
**** P<0.0001
**** P<0.0001
# of foci /cell
100
50
0
Wildtype
H2AX S139A
Δ120-136
Δ120-137
Δ120-138
Δ120-137/A138L
Δ120-137/A138T
d
U2OS H2AX KO + GFP H2A variants (10Gy 1h)
GFP-H2AZ
GFP-MacroH2A1
20µm
MDC1
53BP1
MDC1
53BP1
e
H2AZ
H2AZ-SQEY
H2AZ-SQEL
KDa
25
50
GFP
Tubulin
f
MacroH2A
MacroH2A-SQEY
MacroH2A-SQEL
KDa
50
50
GFP
Tubulin

Supplementary figure legends

Supplementary Figure 1. H2AX has evolved a unique C-terminal tail that is indispensable for DNA repair protein accrual at damaged chromatin

(a) Sequences of H2AX mutants used in Supplementary Fig. 1d and Fig. 1d-f.

(b) Representative immunofluorescence micrographs of ionizing radiation-induced foci for γH2AX and 53BP1 in U2OS H2AX KO cells reconstituted with GFP-empty vector at 1 hour after 10 Gy radiation.

(c) Quantification of total number of 53BP1 foci per cell in mutants reconstituted H2AX KO cells in Fig. 1e and 1f. Foci counts are representative of ≥25 cells from three independent experiments and line representative of the mean. Two-tailed unpaired T-test.

(d) Representative immunofluorescence micrographs of MDC1 and 53BP1 localization in H2AX KO cells expressing GFP-H2AZ or GFP-MacroH2A at 1 hour after 10Gy radiation.

(e) Western blot analysis of H2AZ WT and mutants in reconstituted H2AX KO cells used in figure 2b-c, g-h.

(f) Western blot analysis of macroH2A WT and mutants in reconstituted H2AX KO cells used in figure 2b-c, g-h.

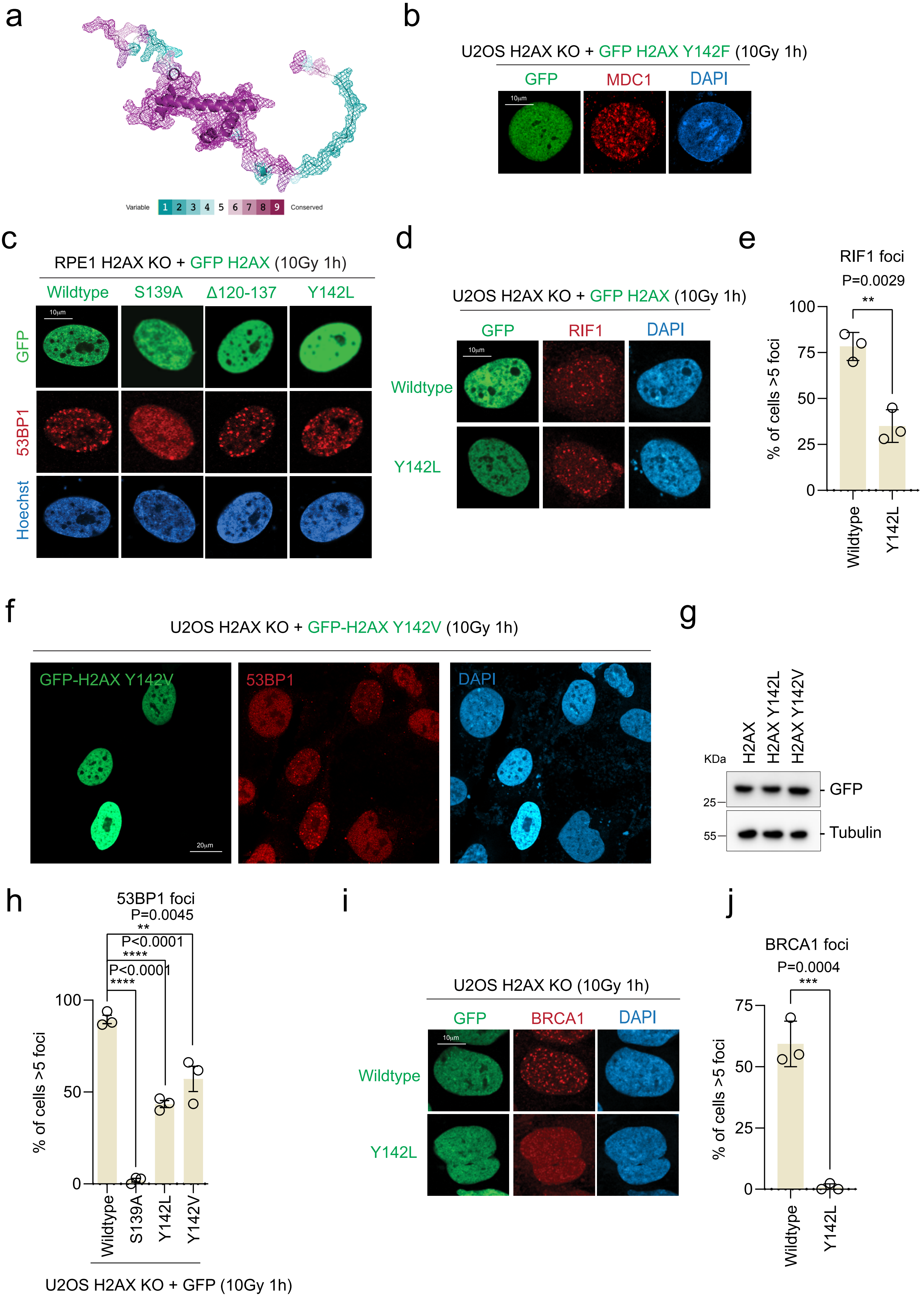
a
Variable
1 2 3 4 5 6 7 8 9
Conserved
b
U2OS H2AX KO + GFP H2AX Y142F (10Gy 1h)
GFP
MDC1
DAPI
10μm
c
RPE1 H2AX KO + GFP H2AX (10Gy 1h)
Wildtype
S139A
Δ120-137
Y142L
GFP
53BP1
Hoechst
10μm
d
U2OS H2AX KO + GFP H2AX (10Gy 1h)
GFP
RIF1
DAPI
Wildtype
Y142L
10μm
e
RIF1 foci
P=0.0029
**
% of cells >5 foci
100
75
50
25
0
Wildtype
Y142L
f
U2OS H2AX KO + GFP-H2AX Y142V (10Gy 1h)
GFP-H2AX Y142V
53BP1
DAPI
20μm
g
KDa
H2AX
H2AX Y142L
H2AX Y142V
GFP
Tubulin
25
55
h
53BP1 foci
P=0.0045
**
P<0.0001
****
P<0.0001
****
% of cells >5 foci
100
50
0
Wildtype
S139A
Y142L
Y142V
U2OS H2AX KO + GFP (10Gy 1h)
i
U2OS H2AX KO (10Gy 1h)
GFP
BRCA1
DAPI
Wildtype
Y142L
10μm
j
BRCA1 foci
P=0.0004
***
% of cells >5 foci
75
50
25
0
Wildtype
Y142L

Supplementary Figure 2. H2AX C-terminal linker mediates MDC1-independent phosphorylation-ubiquitination signaling 53BP1 IRIF formation

(a) Illustration of conservation of H2AX residues in ribbon structure. Evolutionary conservation scores were calculated using ConSurf analysis and illustrated in color as indicated.

(b) Representative immunofluorescence micrographs of ionizing radiation induced foci for MDC1 in H2AX Y142F reconstituted H2AX KO cells at 1 hour after 10 Gy radiation.

(c) Representative immunofluorescence micrographs of 53BP1 localization in RPE1 H2AX KO with H2AX wildtype or mutant reconstitution at 1 hour after 10Gy radiation.

(d) Representative micrographs of RIF1 localization in U2OS H2AX KO with wildtype or Y142L reconstitution at 1 hour after 10 Gy radiation.

(e) Quantification of RIF1 foci as presented in Supplementary Fig. 2d. The error bars correspond to mean±SD of three independent experiments. Two-tailed unpaired T-test.

(f) Representative immunofluorescence micrographs of 53BP1 in H2AX KO with GFP-H2AX Y142V reconstitution at 1 hour after 10Gy radiation.

(g) Western blot analysis of H2AX wildtype and mutant protein levels in H2AX KO reconstituted cells.

(h) Quantification of 53BP1 foci in wildtype and mutants reconstituted H2AX KO cells at 1 hour after 10 Gy radiation. The error bars correspond to mean±SD of three independent experiments. Two-tailed unpaired T-test.

(I) Representative immunofluorescence micrographs of BRCA1 in H2AX KO with GFP-H2AX Y142V reconstitution at 1 hour after 10Gy radiation.

(j) Quantification of BRCA1 foci as presented in Supplementary Fig. 2i. The error bars correspond to mean±SD of three independent experiments. Two-tailed unpaired T-test.

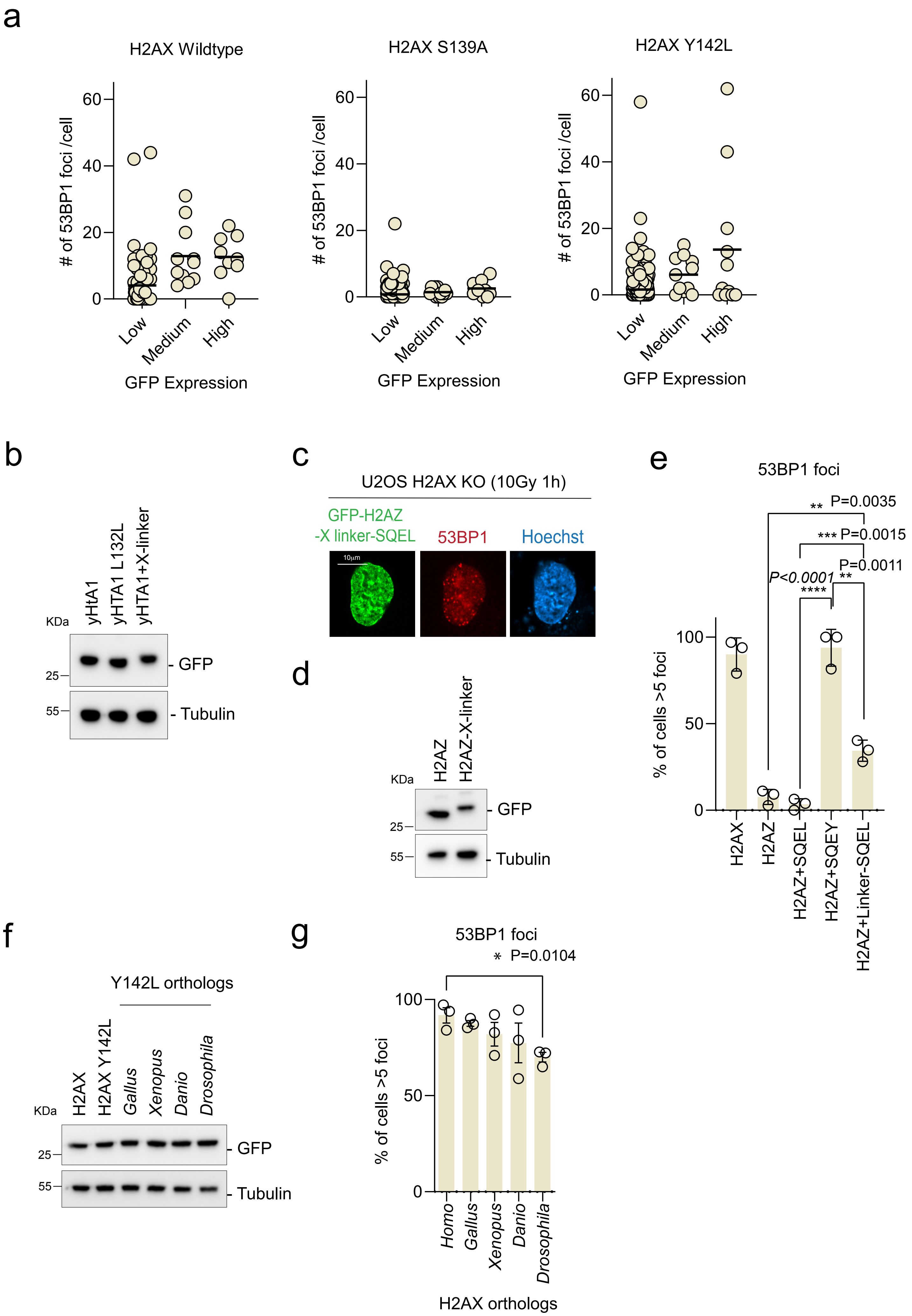

a
H2AX Wildtype
H2AX S139A
H2AX Y142L
# of 53BP1 foci /cell
0
20
40
60
Low
Medium
High
GFP Expression
b
KDa
yHtA1
yHTA1 L132L
yHTA1+X-linker
25
55
- GFP
- Tubulin
c
U2OS H2AX KO (10Gy 1h)
GFP-H2AZ
-X linker-SQEL
53BP1
Hoechst
10µm
d
KDa
H2AZ
H2AZ-X-linker
25
55
- GFP
- Tubulin
e
53BP1 foci
** P=0.0035
*** P=0.0015
P=0.0011
P<0.0001 **
****
% of cells >5 foci
0
50
100
H2AX
H2AZ
H2AZ+SQEL
H2AZ+SQEY
H2AZ+Linker-SQEL
f
Y142L orthologs
KDa
H2AX
H2AX Y142L
Gallus
Xenopus
Danio
Drosophila
25
55
- GFP
- Tubulin
g
53BP1 foci
* P=0.0104
% of cells >5 foci
0
50
100
Homo
Gallus
Xenopus
Danio
Drosophila
H2AX orthologs

Supplementary Figure 3. Molecular dissection of the H2AX C-terminal linker-regulated 53BP1 recruitment

(a) GFP-H2AX expression and 53BP1 foci number binning. The number of 53BP1 foci per cells were quantified and correlates to the levels of GFP-H2AX wildtype or mutants reconstituted H2AX KO cells. Foci counts are representative of ≥25 cells from three independent experiments and line representative of the mean.

(b) Western blot analysis of yHTA1 and mutants used in Fig. 3d.

(c) Representative immunofluorescence micrographs of 53BP1 in H2AX KO with GFP-H2AZ-X-linker-SQEL reconstitution at 1 hour after 10Gy radiation.

(d) Western blot analysis of H2AZ and mutant used in Supplementary Fig. 3c.

(e) Quantification of 53BP1 foci as represented in **Supplementary Fig 3c** and **Fig. 2g**. The error bars correspond to mean ± SD of three independent experiments. Two-tailed unpaired T-test.

(f) Western blot analysis of H2AX Y142L orthologs and mutant expression used in **Fig. 4a-d**.

(g) Quantification of 53BP1 foci in H2AX wildtype orthologs reconstituted H2AX KO cells. The error bars correspond to mean±SD of three independent experiments. Two-tailed unpaired T-test.

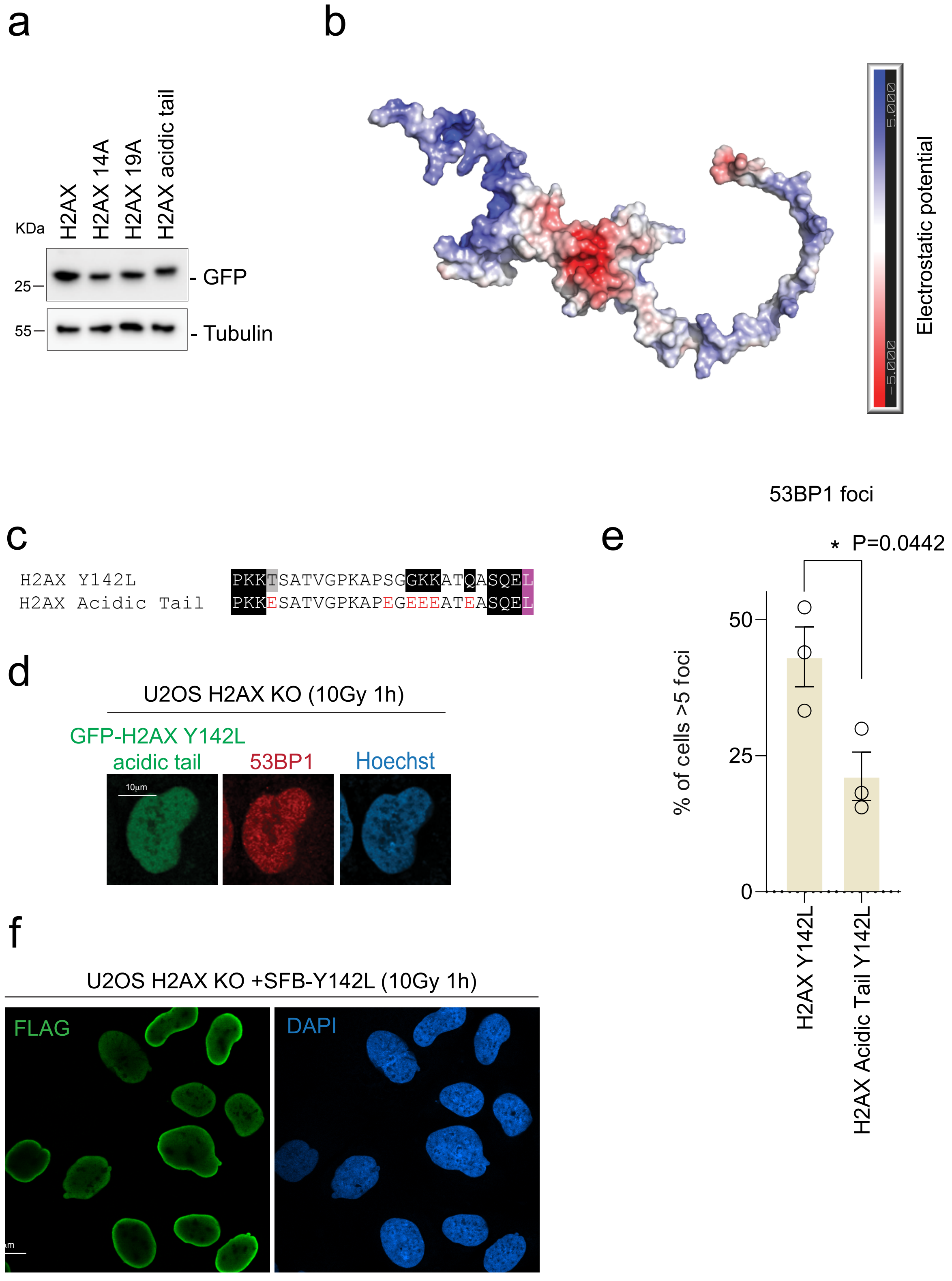

a
KDa
H2AX
H2AX 14A
H2AX 19A
H2AX acidic tail
25
55
GFP
Tubulin
b
5.000
-5.000
Electrostatic potential
c
H2AX Y142L PKKTSATVGPKAPSGGKKATQASQEL
H2AX Acidic Tail PKKESATVGPKAPEGEEEATEASQEL
d
U2OS H2AX KO (10Gy 1h)
GFP-H2AX Y142L acidic tail
53BP1
Hoechst
10µm
e
53BP1 foci
* P=0.0442
% of cells >5 foci
50
25
0
H2AX Y142L
H2AX Acidic Tail Y142L
f
U2OS H2AX KO +SFB-Y142L (10Gy 1h)
FLAG
DAPI

Supplementary Figure 4. Molecular mechanism of the H2AX C-terminal linker region in 53BP1 IRIF formation

(a) Western blot analysis of H2AX mutants expression used in Supplementary 4d and Fig 4e-h.

(b) Electrostatic potential analysis for H2AX.

(c) Alignment of H2AX acidic C-terminal tail Y142L mutant and H2AX Y142L. Conserved residues are highlighted in black. Substituted residues are labelled in red. Leucine substitution highlighted in purple.

(d) Representative immunofluorescence image of 53BP1 in H2AX KO cells reconstituted with GFP-H2AX Y142L acidic tail at 1 hour after 10Gy radiation.

(e) Quantification of 53BP1 foci in H2AX Y142L acidic tail reconstituted H2AX KO cells in Supplementary Fig. 4d. Two-tailed unpaired T-test.

(f) Immunofluorescence image using FLAG antibody in U2OS H2AX KO cells stably expressing SFB-H2AX Y142L.

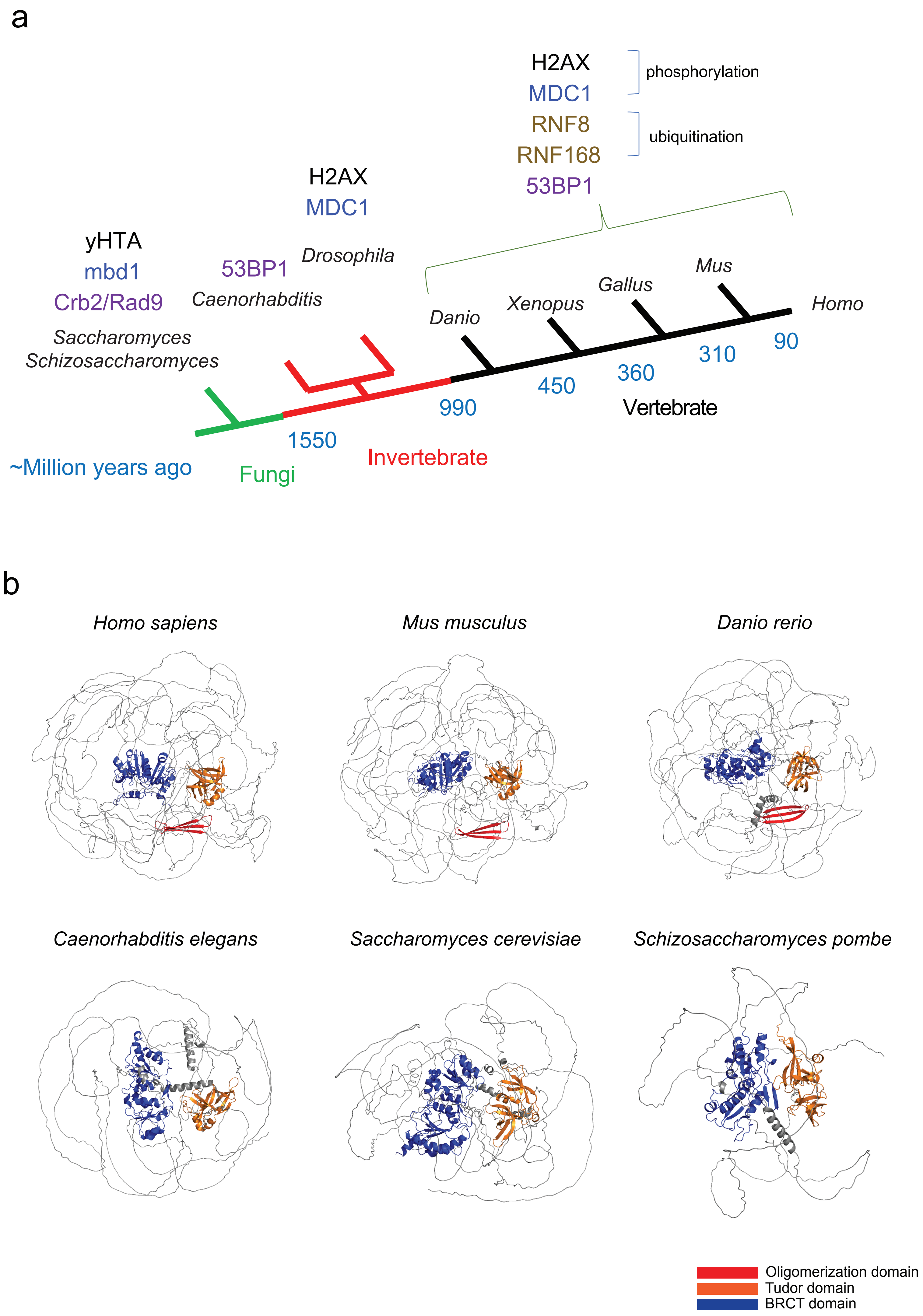

a
H2AX
MDC1
RNF8
RNF168
53BP1
phosphorylation
ubiquitination
H2AX
MDC1
yHTA
mbd1
Crb2/Rad9
53BP1
Drosophila
Caenorhabditis
Saccharomyces
Schizosaccharomyces
Danio
Xenopus
Gallus
Mus
Homo
90
310
360
450
990
1550
Vertebrate
Invertebrate
~Million years ago
Fungi
b
Homo sapiens
Mus musculus
Danio rerio
Caenorhabditis elegans
Saccharomyces cerevisiae
Schizosaccharomyces pombe
Oligomerization domain
Tudor domain
BRCT domain

Supplementary Figure 5. Evolution of the DDR pathway and 53BP1

(a) Phylogenetic representation of the phosphorylation-ubiquitination DDR pathway evolution.

(b) Illustration of 53BP1 homolog structures as predicted by Alphafold. Structural domains were color coded as Red - Oligomerization domain, Orange - Tudor domain, Blue - BRCT domain.

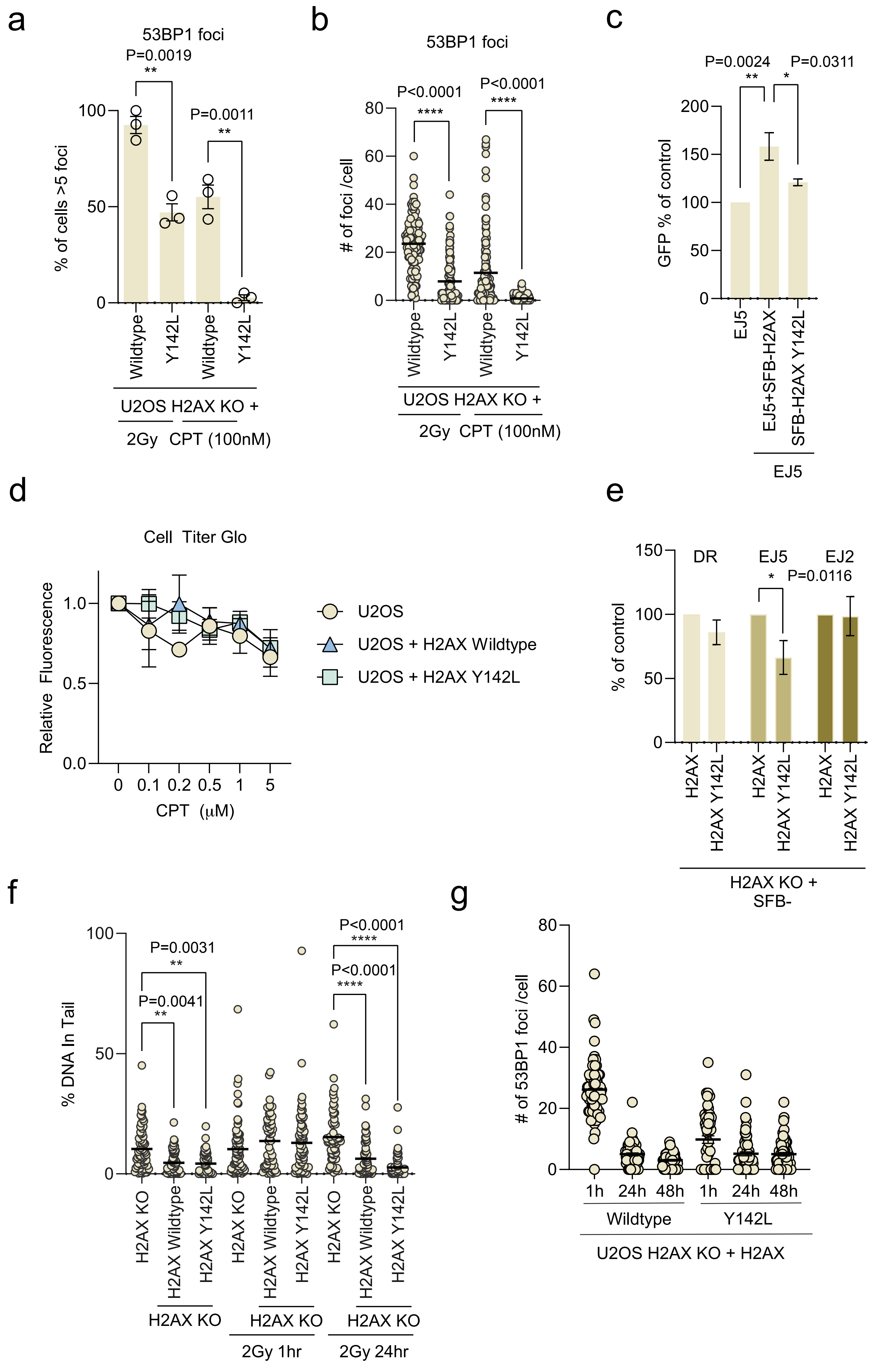
a
53BP1 foci
P=0.0019
**
P=0.0011
**
% of cells >5 foci
100
50
0
Wildtype
Y142L
Wildtype
Y142L
U2OS H2AX KO +
2Gy
CPT (100nM)
b
53BP1 foci
P<0.0001
****
P<0.0001
****
# of foci /cell
80
60
40
20
0
Wildtype
Y142L
Wildtype
Y142L
U2OS H2AX KO +
2Gy
CPT (100nM)
c
P=0.0024
**
P=0.0311
*
GFP % of control
200
150
100
50
0
EJ5
EJ5+SFB-H2AX
SFB-H2AX Y142L
EJ5
d
Cell Titer Glo
Relative Fluorescence
1.0
0.5
0.0
0
0.1
0.2
0.5
1
5
CPT (μM)
U2OS
U2OS + H2AX Wildtype
U2OS + H2AX Y142L
e
150
100
50
0
% of control
DR
EJ5
EJ2
*
P=0.0116
H2AX
H2AX Y142L
H2AX
H2AX Y142L
H2AX
H2AX Y142L
H2AX KO +
SFB-
f
100
50
0
% DNA In Tail
P=0.0031
**
P=0.0041
**
P<0.0001
****
P<0.0001
****
H2AX KO
H2AX Wildtype
H2AX Y142L
H2AX KO
H2AX Wildtype
H2AX Y142L
H2AX KO
H2AX Wildtype
H2AX Y142L
H2AX KO
H2AX KO
H2AX KO
2Gy 1hr
2Gy 24hr
g
80
60
40
20
0
# of 53BP1 foci /cell
1h
24h
48h
1h
24h
48h
Wildtype
Y142L
U2OS H2AX KO + H2AX

Supplementary Figure 6. Functional characterization of the H2AX Y142L mutant

(a) Quantification of 53BP1 foci in H2AX wildtype or Y142L stably reconstituted H2AX KO cells at 1 h after indicated treatments. Data represents Mean±SD from 3 independent experiments. Two-tailed unpaired T-test.

(b) Quantification of 53BP1 foci in H2AX wildtype or Y142L stably reconstituted H2AX KO cells at 1 h after indicated treatments. Foci counts are representative of ≥25 cells from three independent experiments and line representative of the mean. Two-tailed unpaired T-test.

(c) EJ5-GFP reporter cells were electroporated with SFB-H2AX wildtype or SFB-H2AX Y142L mutant using nucleofector. GFP-positive cells were quantified using flow cytometry at 48 hours after transfection. Data represents Mean±SD from 4 independent experiments. Two-tailed unpaired T-test.

(d) Quantification of cell viability in H2AX wildtype or Y142L reconstituted H2AX KO cells using Cell-Titer-Glo assay 5 days after treated with indicated concentration of camptothecin. Data represents Mean±SD from 3 experimental replicates.

(e) H2AX KO with stable H2AX wildtype or H2AX Y142L reconstitution were electroporated with I-SceI vector and reporter vectors: DR-GFP, EJ5-GFP, EJ2-GFP. GFP-positive cells were quantified using flow cytometry at 48 hours after transfection. Data represents Mean±SD from 3 independent experiments.

(f) Quantification of percentage of DNA in tail for alkaline comets after 2Gy irradiation at indicated time points. Counts are representative of ≥25 cells from three independent experiments and line representative of the mean. One-way ANOVA.

(g) Quantification of 53BP1 foci per cell in U2OS H2AX KO cells reconstituted with H2AX WT or Y142L at the indicated timepoints after 2Gy irradiation. Foci counts are representative of ≥25 cells from three independent experiments and line representative of the mean.

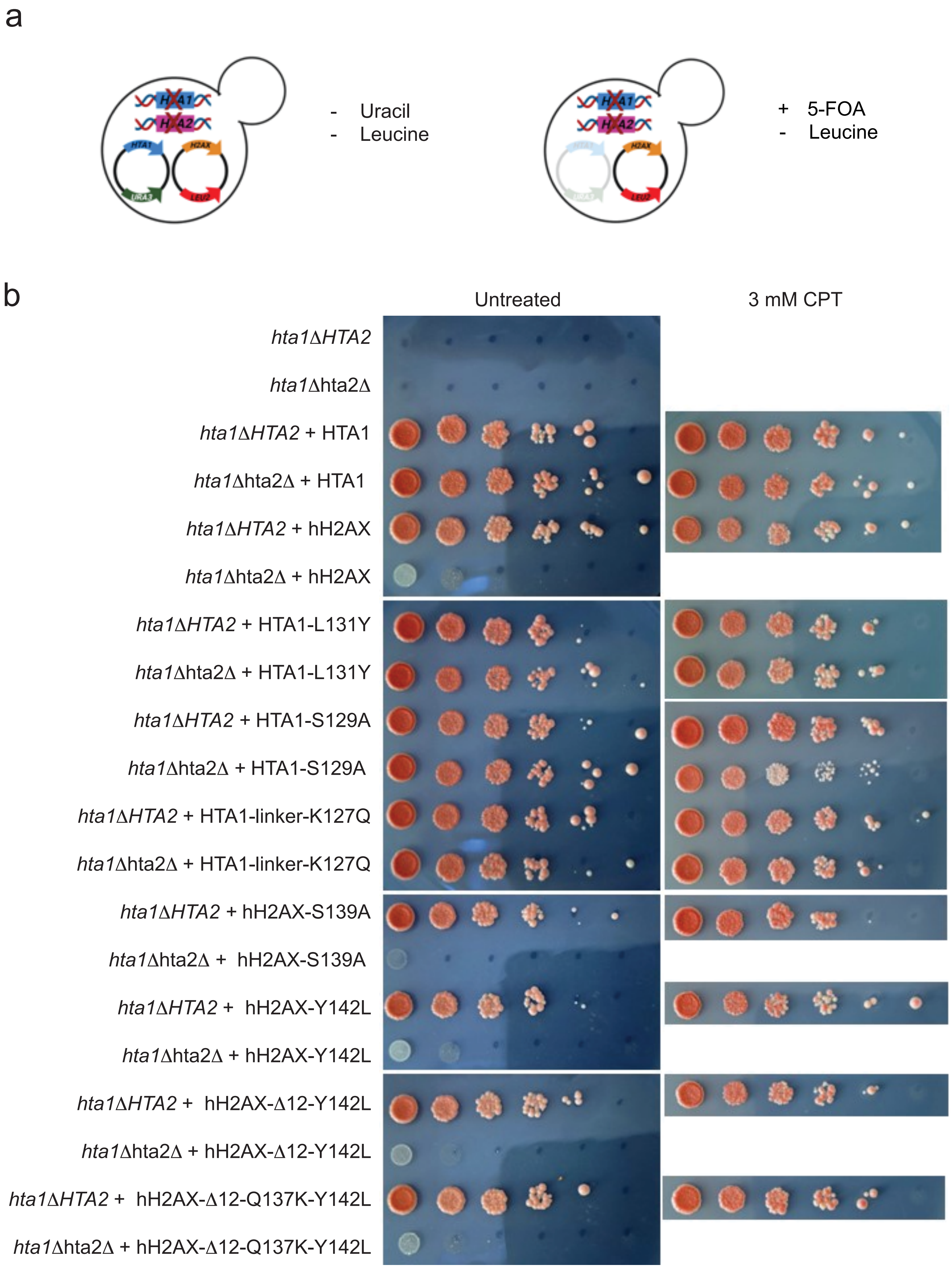
a
HTA1
HTA2
H2AX
URA3
LEU2
- Uracil
- Leucine
+ 5-FOA
- Leucine
b
Untreated
3 mM CPT
hta1ΔHTA2
hta1Δhta2Δ
hta1ΔHTA2 + HTA1
hta1Δhta2Δ + HTA1
hta1ΔHTA2 + hH2AX
hta1Δhta2Δ + hH2AX
hta1ΔHTA2 + HTA1-L131Y
hta1Δhta2Δ + HTA1-L131Y
hta1ΔHTA2 + HTA1-S129A
hta1Δhta2Δ + HTA1-S129A
hta1ΔHTA2 + HTA1-linker-K127Q
hta1Δhta2Δ + HTA1-linker-K127Q
hta1ΔHTA2 + hH2AX-S139A
hta1Δhta2Δ + hH2AX-S139A
hta1ΔHTA2 + hH2AX-Y142L
hta1Δhta2Δ + hH2AX-Y142L
hta1ΔHTA2 + hH2AX-Δ12-Y142L
hta1Δhta2Δ + hH2AX-Δ12-Y142L
hta1ΔHTA2 + hH2AX-Δ12-Q137K-Y142L
hta1Δhta2Δ + hH2AX-Δ12-Q137K-Y142L

Supplementary Figure 7: Human H2AX is functionally different from yeast HTA

(a) Yeast plasmid shuttle assay. Yeast *hta1Δ* and *hta2Δ* mutants carry a *URA3*-marked plasmid expressing *HTA1* and a *LEU2*-marked plasmid expressing the complementing yeast HTA or human H2AX mutants to be tested. Cells are grown in liquid medium lacking uracil and leucine. Cells are then spotted onto plates lacking leucine and supplemented with 5-fluoroorotic acid (5-FOA) to select against cells with the *URA3*-marked *HTA1* plasmid. Created with BioRender.com released under a Creative Commons Attribution-NonCommercial-NoDerivs 4.0 International license.

(b) Mutants with the indicated genotype and with a *LEU2*-bearing plasmid expressing the indicated *HTA1* or *H2AX* variant were spotted to either -leucine +5FOA or -leucine +5FOA + 3mM camptothecin (CPT) plates and incubated for 4 days at 30ºC prior to being photographed. Spots are 5μL, 10-fold serial dilutions with an equal number of cells for each condition determined by optical density.
