# Supplementary material for: Evolved histone tail regulates 53BP1 recruitment at damaged chromatin
Source: Nat Commun. 2024 May 31;15:4634. doi: 10.1038/s41467-024-49071-w (PMC11143218; doi:10.1038/s41467-024-49071-w)
Supplement: Supplementary file 3 — Description of Additional Supplementary Files [file 41467_2024_49071_MOESM3_ESM.pdf]

Description of Additional Supplementary Files

File Name: Supplementary Data 1
Description: Primers and geneblocks sequence used in current study
