# Supplementary material for: Evolved histone tail regulates 53BP1 recruitment at damaged chromatin
Source: Nat Commun. 2024 May 31;15:4634. doi: 10.1038/s41467-024-49071-w (PMC11143218; doi:10.1038/s41467-024-49071-w)
Supplement: Supplementary file 5 — Reporting Summary [file 41467_2024_49071_MOESM5_ESM.pdf]

nature portfolio

Corresponding author(s): Justin Leung

Last updated by author(s): Apr 26, 2024

Reporting Summary

Nature Portfolio wishes to improve the reproducibility of the work that we publish. This form provides structure for consistency and transparency in reporting. For further information on Nature Portfolio policies, see our Editorial Policies and the Editorial Policy Checklist.

Statistics

For all statistical analyses, confirm that the following items are present in the figure legend, table legend, main text, or Methods section.

n/a	Confirmed	
☒	☐	The exact sample size (*n*) for each experimental group/condition, given as a discrete number and unit of measurement
☐	☒	A statement on whether measurements were taken from distinct samples or whether the same sample was measured repeatedly
☐	☒	The statistical test(s) used AND whether they are one- or two-sided<br>*Only common tests should be described solely by name; describe more complex techniques in the Methods section.*
☒	☐	A description of all covariates tested
☐	☒	A description of any assumptions or corrections, such as tests of normality and adjustment for multiple comparisons
☐	☒	A full description of the statistical parameters including central tendency (e.g. means) or other basic estimates (e.g. regression coefficient) AND variation (e.g. standard deviation) or associated estimates of uncertainty (e.g. confidence intervals)
☐	☒	For null hypothesis testing, the test statistic (e.g. F, t, r) with confidence intervals, effect sizes, degrees of freedom and P value noted<br>*Give P values as exact values whenever suitable.*
☒	☐	For Bayesian analysis, information on the choice of priors and Markov chain Monte Carlo settings
☒	☐	For hierarchical and complex designs, identification of the appropriate level for tests and full reporting of outcomes
☒	☐	Estimates of effect sizes (e.g. Cohen's *d*, Pearson's *r*), indicating how they were calculated

Our web collection on statistics for biologists contains articles on many of the points above.

Software and code

Policy information about availability of computer code

Data collection	no software was used for data collection
Data analysis	Pymol, GraphPad Prism, Clustal Omega, Boxshade

For manuscripts utilizing custom algorithms or software that are central to the research but not yet described in published literature, software must be made available to editors and reviewers. We strongly encourage code deposition in a community repository (e.g. GitHub). See the Nature Portfolio guidelines for submitting code & software for further information.

Data

Policy information about availability of data

All manuscripts must include a data availability statement. This statement should provide the following information, where applicable:

- Accession codes, unique identifiers, or web links for publicly available datasets
- A description of any restrictions on data availability
- For clinical datasets or third party data, please ensure that the statement adheres to our policy

All data are available from the corresponding author upon reasonable request

Research involving human participants, their data, or biological material

Policy information about studies with human participants or human data. See also policy information about sex, gender (identity/presentation), and sexual orientation and race, ethnicity and racism.

Reporting on sex and gender	NA
Reporting on race, ethnicity, or other socially relevant groupings	NA
Population characteristics	NA
Recruitment	NA
Ethics oversight	NA

Note that full information on the approval of the study protocol must also be provided in the manuscript.

Field-specific reporting

Please select the one below that is the best fit for your research. If you are not sure, read the appropriate sections before making your selection.

☒ Life sciences ☐ Behavioural & social sciences ☐ Ecological, evolutionary & environmental sciences

For a reference copy of the document with all sections, see nature.com/documents/nr-reporting-summary-flat.pdf

Life sciences study design

All studies must disclose on these points even when the disclosure is negative.

Sample size	Sample sizes were as large as possible given the experimental system used with at least three independent replicates
Data exclusions	None
Replication	All experiments were carried out independently for at least three times
Randomization	Randomization was not necessary, the appropriate controls were used
Blinding	Data collection and Analysis were not performed blindly, we have proper internal controls for every single experiment.

Reporting for specific materials, systems and methods

We require information from authors about some types of materials, experimental systems and methods used in many studies. Here, indicate whether each material, system or method listed is relevant to your study. If you are not sure if a list item applies to your research, read the appropriate section before selecting a response.

Materials & experimental systems

n/a	Involved in the study	
☐	☒	Antibodies
☐	☒	Eukaryotic cell lines
☒	☐	Palaeontology and archaeology
☒	☐	Animals and other organisms
☒	☐	Clinical data
☒	☐	Dual use research of concern
☒	☐	Plants

Methods

n/a	Involved in the study	
☒	☐	ChIP-seq
☐	☒	Flow cytometry
☒	☐	MRI-based neuroimaging

Antibodies

Antibodies used	GFP (Invitrogen, A11122)<br>mouse monoclonal γH2AX (JBW301) (EMD Millipore, 05-636),<br>rabbit polyclonal γH2AX (Cell Signaling, 2577L),<br>mouse monoclonal Flag (M2) (Sigma, F1804),

mouse monoclonal c-myc (9E10) (Santa Cruz sc-40),
rabbit monoclonal MBP (EPR4744) (Abcam ab119994),
mouse monoclonal beta tubulin (Santa Cruz, sc-166729),
53BP1 (Novus Biologicals, NB100-304),
mouse monoclonal BRCA1 (Santa Cruz, sc6954)
mouse monoclonal RIF1 (Santa Cruz, sc-515573)
HRP-linked anti-rabbit IgG (Jackson ImmunoResearch 115-035-144)
HRP-linked anti-mouse IgG (Jackson ImmunoResearch 115-035-166)
Alexa Fluor 488 goat anti-rabbit (Invitrogen, A11034)
Alexia Fluor 594 goat antimouse (Invirtogen, A11037)

Validation

GFP (Invitrogen, A11122), validation based on manufacturer's data sheet by wester blotting, validation reference PMID: 29718323
γH2AX (Millipore, 05-636), validation reference PMID: 28242625
rabbit polyclonal γH2AX (Cell Signaling, 2577L), validation based on manufacturer data sheet, validation reference PMID: 28943310
Flag M2 (Sigma, F1804), validation reference PMID: 28242625
c-Myc (Santa Cruz, sc-40), validation reference PMID: 28242625
rabbit monoclonal MBP (EPR4744) (Abcam ab119994),validation based on manufacturer's data sheet
mouse monoclonal beta tubulin (Santa Cruz, sc-166729), validation cased on manufacturer's data sheet
53BP1 (Novus Biologicals, NB100-304), validation reference PMID: 28242625
mouse monoclonal BRCA1 (Santa Cruz, sc6954), validation reference PMID: 32424115
mouse monoclonal RIF1 (Santa Cruz, sc-515573)validation based on manufacturer's data sheet
HRP-linked anti-rabbit IgG (Cell signaling, 0704) validation based on manufacturer's data sheet
HRP-linked anti-mouse IgG (Cell signaling, 0706) validation based on manufacturer's data sheet
Alexa Fluor 488 goat anti-rabbit (Invitrogen, A11034) validation based on manufacturer's data sheet
Alexia Fluor 594 goat antimouse (Invirtogen, A11037) validation based on manufacturer's data sheet

Eukaryotic cell lines

Policy information about cell lines and Sex and Gender in Research

Cell line source(s)

ATCC: HEK293T (CRL-3216), U2OS (HTB-96), U2OS H2AX
Steve Jackson's lab: U2OS H2AX KO and RPE1 H2AX KO PMID: 31729360

Authentication

None of the cell lines used were authenticated

Mycoplasma contamination

All cell lines were tested negative for mycoplasma contamination

Commonly misidentified lines
(See ICLAC register)

No commonly misidentified cell lines were used

Plants

Seed stocks

NA

Novel plant genotypes

NA

Authentication

NA

Flow Cytometry

Plots

Confirm that:

☐ The axis labels state the marker and fluorochrome used (e.g. CD4-FITC).

☐ The axis scales are clearly visible. Include numbers along axes only for bottom left plot of group (a 'group' is an analysis of identical markers).

☐ All plots are contour plots with outliers or pseudocolor plots.

☒ A numerical value for number of cells or percentage (with statistics) is provided.

Methodology

Sample preparation

48 hours after electroporation were trypsinized and harvested in PBS with 2% BSA

Instrument	BD Accuri C6 plus
Software	BD Accuri C6 plus analysis software
Cell population abundance	30000 cells per analysis
Gating strategy	For the repair pathway choice repair assays, we sort the GFP-positive cells using two gates in the following plots. 1) SSC-H scatter (Y-axis) set out against FSC-H scatter (X-axis), allowing us to set gate P1, and 2) SSC-H scatter (Y-axis) set out against FITC-H scatter (X-axis) allowing us to set gate P2. GFP positive cells were scored using a gating based on GFP-negative control cells.

☐ Tick this box to confirm that a figure exemplifying the gating strategy is provided in the Supplementary Information.
